# Meldonium Ameliorates Hypoxia-Induced Lung Injury and Oxidative Stress by Regulating Platelet-Type Phosphofructokinase-Mediated Glycolysis

**DOI:** 10.3389/fphar.2022.863451

**Published:** 2022-04-05

**Authors:** Daohui Wang, Fengying Liu, Weijie Yang, Yangyang Sun, Xiaoning Wang, Xin Sui, Jun Yang, Qian Wang, Wenhao Song, Minmin Zhang, Zhenyu Xiao, Tian Wang, Yongan Wang, Yuan Luo

**Affiliations:** ^1^ State Key Laboratory of Toxicology and Medical Countermeasures, Beijing Institute of Pharmacology and Toxicology, Beijing, China; ^2^ School of Pharmacy, Key Laboratory of Molecular Pharmacology and Drug Evaluation, Ministry of Education (Yantai University), Yantai University, Yantai, China-

**Keywords:** PFKP, lung injury, high altitude, hypoxia, oxidative stress, mitochondrion, glycolysis, meldonium

## Abstract

Hypoxic environments at high altitudes influence the long-term non-altitude health of residents, by inducing changes in metabolism and the mitochondria, severe lung injury, and endangering life. This study was aimed to determine whether meldonium can ameliorate hypoxia-induced lung injury and investigate its possible molecular mechanisms. We used Swiss mice and exposed type Ⅱ alveolar epithelial cell to hypobaric hypoxic conditions to induce lung injury and found that meldonium has significant preventive effect, which was associated with the regulation of glycolysis. We found using human proteome microarrays assay, molecular docking, immunofluorescence and pull-down assay that the target protein of meldonium is a platelet-type phosphofructokinase (PFKP), which is a rate-limiting enzyme of glycolysis. Also, meldonium promotes the transfer of nuclear factor erythroid 2-related factor 2 (Nrf2) from the cytoplasm to the nucleus, which mitigates oxidative stress and mitochondrial damage under hypoxic condition. Mechanistically, meldonium ameliorates lung injury by targeting PFKP to regulate glycolysis, which promotes Nrf2 translocation from the cytoplasm to the nucleus to alleviate oxidative stress and mitochondrial damage under hypoxic condition. Our study provides a novel potential prevention and treatment strategy against hypoxia-induced lung injury.

## Introduction

High altitude refers to regions more than 2,500 m above sea level. With the development of transportation and tourism in high-altitude locations, an increasing number of individuals travel to high-altitude regions for travel or work, and they primarily include mountaineers, explorers, and geoexplorers. However, due to the complexity and variability of climate and topography, hypobaric hypoxic environments at high altitudes not only influence work efficiency, life experience, and medical treatment efficiency, but also elicit distinct pathological reactions to non-altitude long-term residents (Bärtsch and Swenson, 2013). Acute mountain sickness (AMS) occurs at high altitudes, and high-altitude pulmonary oedema (HAPE) and high-altitude cerebral oedema (HACE) can progress without timely treatment, endangering lives (Wang et al., 2016a; Wang et al., 2016b). Common accompanying initial symptoms are headache, weakness, and decreased consciousness (Davis and Hackett, 2017); notably, hypobaric hypoxia induces severe lung injury (Boos et al., 2012).

Under normoxic conditions, the hypoxia-inducible factor-1α (HIF-1α) is hydroxylated by proline hydroxylase (PHD), ubiquitinated by Von Hippel Lindau, and ultimately degraded by the proteasome. However, the activity of PHD is inhibited under low oxygen conditions, which induces the stability of HIF-1α. HIF-1α dimerises with HIF-1β to form HIF-1 and enters the nucleus. Subsequently, HIF-1 activates gene transcription by binding to the hypoxia response elements (HRE) at the promoter region, thereby regulating metabolism and lung injury induced by hypoxia (Gerald et al., 2004). These genes include inflammatory factors and glycolytic enzymes that regulate acute lung injury in hypoxic environments (Suresh et al., 2014). The cascade of inflammation is important in the formation of hypoxia-induced lung injury (Salama et al., 2014). HIF-1α inhibition ameliorated the inflammatory cascade to alleviate injury induced by lung contusion (Suresh et al., 2014). However, the mechanism of regulation of lung injury through glycolysis remains unclear. Besides, oxidative stress occurs when subjects are exposed to high altitude, which is considered the main factor of hypoxia-induced lung injury (Gong et al., 2018). Hypoxia induces abnormality in complex III of the mitochondrial electron transport chain and produces reactive oxygen species (ROS) that can induce tissue damage (Nordberg and Arnér, 2001; Jung et al., 2010). However, the molecular mechanisms underpinning such high-altitude-induced injuries remain unclear, limiting the development of effective treatments.

To date, many prevention and treatment measures have been used against injury induced by hypobaric hypoxia at high altitudes, drugs being the main method (Wang et al., 2016b). Acetazolamide is the only drug approved by the U.S. Food and Drug Administration (FDA) to prevent AMS, which improves pulmonary gas circulation and blood oxygen saturation (Jonk et al., 2007). Acetazolamide alleviates the degree of multiple organ injury in rats subjected to hypobaric hypoxia by reducing the expression of pro-inflammatory factors (Wang et al., 2016a). Dexamethasone, which clinically used for myocardial protection and anti-oxidative stress, can also alleviate lung injury induced by environmental interference and restore respiratory function (Kaur and Singh, 2011; Tang and Xia, 2017). However, side effects such as nausea, vomiting, and electrolyte disturbances, affect the patient’s quality of life and treatment efficacy at high altitudes (Inatani et al., 1999). Thus, it is necessary to explore drugs that have preventive effects under the special conditions prevalent at high altitudes.

Meldonium, a structural analogue of carnitine, was originally developed by the Latvian Institute of Organic Synthesis in the 1970s. It has significant pharmacological activity in ameliorating cardiovascular diseases, metabolic syndromes, and diabetes. Mechanistically, meldonium reduces the content of L-carnitine by inhibiting γ-butyrobetaine hydroxylase, carnitine/organic cation transporter type 2 (OCTN2), and competitively binds carnitine/acylcarnitine translocase at the same site. Moreover, the levels of long-chain acylcarnitines, products generated from L-carnitine catalysed by carnitine palmitoyltransferase-1 (CPT-1), transported into the mitochondria were decreased, thus changing the metabolic patterns from fatty acid oxidation to glucose metabolism and providing protection against cardiovascular diseases (Wang and Lopaschuk, 2007; Klusa et al., 2013). Thus, L-carnitine homeostasis is an important target for regulating energy metabolism pathways and treating metabolic diseases (Dambrova et al., 2016). A previous clinical study shows that meldonium improves the exercise capacity of athletes (Kakhabrishvili et al., 2002). Previous studies have demonstrated that meldonium enhances the aerobic oxidation of glucose, decreases lactate content in cardiomyocytes (Liepinsh et al., 2008), reduces the use of pyruvate for metabolism in cells (Makrecka et al., 2014), and decreases lactate concentration in the blood of obese rats (Liepinsh et al., 2011). More importantly, prolonged meldonium treatment optimises energy metabolism during hypoxia to maintain ATP production (Liepinsh et al., 2013). These reports suggested that meldonium has the ability to regulate glucose metabolism and even glycolysis, but the mechanism was need further study.

A previous study has indicated that hypoxia results in metabolic disorders, which cause multiple organ dysfunction and damage, especially the respiratory system (Chang et al., 2019). Hypoxia elevates glycolysis, a pivotal energy metabolic pathway regulated by various enzymes (Garcia-Alvarez et al., 2014). Among them, phosphofructokinase 1 (PFK1), a second rate-limiting enzyme in glycolysis, is the major regulate point. It has three subtypes, of which PFKP, the platelet isoform of phosphofructokinase, is the most widely distributed. Thus, we investigate the protective effects of meldonium on lung injury induced by hypobaric hypoxia and discuss the potential mechanisms involved in glycolysis regulation and mitochondrial protection by meldonium.

## Materials and Methods

### Chemicals and Reagents

Meldonium was synthesised and provided by Yuanye Biological Technology Co., Ltd. (Shanghai, China). It was diluted in sterilised water for *in vivo* experiments, dissolved in normal saline, and diluted with RPMI-1640 medium for *in vitro* experiments. Acetazolamide was purchased from Selleck Chemicals (Batch No. S4506. Houston, United States) and was dissolved in sterilised water containing 40% cyclodextrins and 1% dimethyl sulfoxide (Sinopharm, Shanghai, China) and diluted with RPMI-1640 medium. Biotin-labelled meldonium was prepared by Wayen Biological Technology Co., Ltd. (Shanghai, China). Biotin was obtained from Invitrogen Co., Ltd. (Massachusetts, United States). Biotin-labelled meldonium and biotin were diluted in RPMI-1640 medium.

### Animals and Experimental Design

Adult male Swiss mice weighing 20 ± 2 g were provided by Beijing SiPeifu Biotech Co., Ltd. (Beijing, China). Mice were treated humanely and maintained in a temperature-controlled room (22 ± 2°C) with a 12-h light/dark cycle (lights on at 07:00 and off at 19:00); food and water were available *ad libitum*. All animal experiments were conducted in accordance with the national legislation and approved by the Institutional Animal Care and Use Committee (IACUC number: IACUC-DWZX-2021-762, Laboratory Animal Center of the Academy of Military Medical Science, Beijing, China).

Mice were divided randomly into six groups: 1) Control group: physiological saline, 2) Hypoxia group: Mice were pre-treated with physiological saline before being placed into a hypobaric hypoxia experimental chamber to simulate acute exposure to high altitude (Guizhou Fenglei aero armoury Co., Ltd., Guizhou, China). The chamber conditions were as follows: equivalent height above sea level, 8,000 m; operating time, 24 h; and simulated lifting speed, 30 m/s. (3–5) Meldonium groups: Mice were pre-treated with different concentrations of meldonium (50, 100, or 200 mg/kg) before exposure to hypobaric hypoxia. 6) Acetazolamide group: Mice were pre-treated with acetazolamide (50 mg/kg), which was used as a positive control before exposure to hypobaric hypoxia. Meldonium and acetazolamide were administered once daily for 3 days. Following the induction of hypoxia for 24 h, mice were sacrificed by euthanasia for experiments. This study was performed in accordance with the guidelines for the care and use of laboratory animals, of the National Institute of Health, United States (Guide for the Care and Use of Laboratory Animals, 2011).

### Blood Gas Analysis

Twenty-four hours after hypoxia exposure, the animals were anaesthetised with ketamine (90 mg/kg) and xylazine (5 mg/kg) (intraperitoneal injection). Blood was collected from the abdominal aorta of mice and analysed using an automated blood gas analyser (Radiometer, Copenhagen, Denmark). Blood pH, partial pressure of carbon dioxide (pCO_2_), and bicarbonate ion concentration (cHCO_3_^−^) were recorded.

### Histomorphology

After anaesthetisation, mice were perfused with 4% paraformaldehyde. Lung tissue was collected and fixed in 4% paraformaldehyde overnight and cut into 4 μm thick sections for haematoxylin and eosin (H&E) staining. The pathological changes were evaluated by three pathologists, who were blinded to the design, using an inverted microscope (IX71, Olympus, Tokyo, Japan). Lung injury was detected using the Smith scoring criteria: pulmonary hyaline membrane formation, alveolar cavity enlargement, alveolar wall thickening, haemorrhage, necrosis, and inflammatory cell infiltration. Lung injury severity was graded from 0 to 4.

### Measurement of Superoxide Dismutase and Malondialdehyde Levels

Lung tissue (30 mg) was mixed with cold normal saline at a ratio of 1:10 (weight to volume), and homogenised by using a high-speed cryogenic tissue grinder (Servicebio, Wuhan, China). SOD activity and MDA content in the lung were measured using commercial kits (Jiancheng Bioengineering Institute, Nanjing, China) according to the manufacturer’s instructions. MDA content was measured at 532 nm, while SOD activity was 450 nm.

### Cell Culture and Experimental Design

Rat alveolar type II epithelial RLE-6TN cells (CRL-2300, ATCC, Virginia, United States) were cultured in RPMI-1640 medium (Gibco, California, United States) supplemented with 10% foetal bovine serum (HyClone, Logan, Utah, United States). Cells were seeded into cell culture plates at a density of 5 × 10^4^ cells/mL. The cells were divided into six groups: 1) Control group and 2) Hypoxia group, cells were cultured with normal complete culture medium; (3–5) Meldonium groups, complete culture medium containing 10, 20, or 40 μM meldonium, respectively; 6) Acetazolamide group, complete culture medium containing 10 μM acetazolamide. After culturing in a 5% CO_2_ incubator at 37°C with saturated humidity for 24 h, except of the cells in the control group, other groups were placed in a humidified 37°C hypoxia incubator (Baker Ruskinn, United States) for 24 h. The conditions of the hypoxia incubator were as follows: 0.5% O_2_, 5% CO_2_, supplemented with N_2_.

### Cell Viability Assay

Cell viability was assayed using the Cell Counting Kit-8 (CCK-8, Dojindo, Kumamoto Prefecture, Japan) according to the manufacturer’s protocol. The optical density of the samples was measured at 450 nm using a microplate reader (Molecular Devices, California, United States).

### Measurement of Lactate

The lactate concentration in the supernatant of the cell culture medium was quantified using a Lactate Assay Kit (I256, Dojindo, Kumamoto Prefecture, Japan) according to the manufacturer’s protocol. The absorbance of the sample was measured at 450 nm.

### Metabolic Assay

Oxygen consumption rate (OCR) and extracellular acidification rate (ECAR) represent the functions of mitochondria and glycolysis. Metabolic changes in the cells were analysed using a Seahorse XFe96 Extracellular Flux Analyser (Agilent Technologies, California, United States). 4×10^3^ cells per well were seeded into an XF 96 cell culture microplate (Agilent Technologies, California, United States). OCR and ECAR were assayed using the Seahorse XF Cell Mito Stress Test Kit (Agilent Technologies, California, United States, 103015–100) and Seahorse XF Glycolysis Stress Test Kit (Agilent Technologies, California, United States, 103020–100). Inhibitors and activators were used per well at the following concentrations *via* ports A–C: 1) ECAR including glucose (10 mM), oligomycin (1 μM) and 2-DG (50 mM); 2) OCR including oligomycin (1.5 μM), FCCP (0.5 μM) and Rot/AA (0.5 μM). Data were assessed using the Seahorse XF96 Wave software (Agilent Technologies, California, United States).

### Human Proteome Microarray Analysis and Data Processing

The HuProt™ human proteome microarray, composed of approximately 20,000 purified human full-length proteins with N-terminal glutathione S-transferase tags, was provided by Wayen Biotechnology (Shanghai, China) to investigate the target proteins of meldonium. First, the microarray was incubated with blocking buffer (5% bovine serum albumin in 1× PBST) at 4°C in the dark for 1.5 h. Microarrays were then incubated with 1 μM biotin-meldonium or 1 μM biotin at 37°C for 1 h. After washing three times with PBST and twice with distilled water, Cy5-Streptavidin solution (1:1,000 dilution) was added and incubated at 37°C for 20 min. Then, the microarray was washed with PBST 0.1 × PBS and subsequently distilled water and spun dry at 1,000 rpm for 2 min. A GenePix 4000 microarray scanner (Axon Instruments, California, United States) was used to visualise and record the results, and GenePix™ Pro v6.0 (Axon Instruments, California, United States) was used for data analysis.

The candidate positive proteins were identified by the Z-Score of protein spot (more than 2.8 in the biotin-meldonium microarray and less than 2.8 in the biotin microarray). Next, the candidate positive protein was determined as the final meldonium target protein when the Imean-Ratio was more than 1.4, obtained by calculating the ratio of Z-Score between biotin-meldonium microarray and biotin microarray. The enrichment analysis of target protein candidates, including the Kyoto Encyclopedia of Genes and Genomes (KEGG) pathway, biological process, molecular function, and cellular component, was performed using the Database for Annotation, Visualization, and Integrated Discovery (DAVID) v6.7.

### Molecular Docking

Molecular docking was employed to verify the binding energy between meldonium and the target protein PFKP. The protein crystal structure and PDB format file of PFKP with PDBID 4XYK were downloaded from The PDB Protein Database (http://www.rcsb.org/pdb/home.do). The structure of meldonium was downloaded from The Database of The Chemical Book (http://www.chemicalbook.cn). The target protein and compound molecule were hydrogenated, charged, and optimised using MOE software (Molecular Operating Environment, 10.2008, chemical computing group). After structural optimisation, the ligand interacts with the protein receptor. Ligand-receptor binding was evaluated using the ASE function score value. Low binding energy represents a strong ligand-receptor affinity.

### Immunofluorescence Analysis

RLE-6TN cells were seeded in a plate with a glass bottom (Cellvis, Maryland, United States) and divided into four groups: 1) Negative control group: normal complete culture medium; 2) Meldonium group: complete culture medium containing 100 μM meldonium; 3) Biotin-labelled meldonium group, complete culture medium containing 100 μM biotin-labelled meldonium; 4) Biotin group: complete culture medium containing biotin. After incubation for 24 h, the cells were fixed with 4% paraformaldehyde for 30 min and washed with cold PBS three times. Cells were permeabilised with 0.2% Triton X-100 at 4°C for 10 min and blocked with 5% fat-free milk for 30 min at room temperature, followed by incubation with the primary antibody PFKP (1:300, Abcam, Cambridge, United Kingdom) at 4°C overnight. The samples were then incubated with Alexa Fluor^®^ 488 donkey anti-rabbit IgG secondary antibody (1:1,000, ab150061, Abcam, Cambridge, United Kingdom) at room temperature for 1 h in the dark. The cells were incubated with streptomycin (1:1,000, Thermo Fisher Scientific, Massachusetts, United States) at room temperature for 2 h, and then stained with Hoechst 33342 (1:1,000, H3570, Invitrogen, California, United States) at room temperature for 5 min in the dark. Microphotographs were observed using ImageXpress Micro Confocal (Molecular Devices, California, United States) by a colleague blinded to the experimental design. The cells co-localised with biotin-labelled meldonium (red) and anti-PFKP antibody-labelled PFKP (green) appeared yellow or orange.

### Pull-Down Assay

RLE-6TN cells in 6-well cell culture plates at a density of 2×10^7^ cells/ml were collected and lysed using RIPA buffer (Thermo Fisher Scientific, Massachusetts, United States) containing a protease inhibitor cocktail (Bimake, Houston, United States), centrifuged at 14,000 × *g* for 15 min at 4°C. The supernatant was added to centrifugal filters (Millipore, Massachusetts, United States) and centrifuged at 4,000 × *g* for 40 min at 4°C. Biotin-meldonium or biotin (200 μl, 10 μM) was added to the supernatant. To allow meldonium to bind the protein, the supernatant was rotated overnight at 4°C. Streptavidin-agarose beads (150 μl, Invitrogen, California, United States) were added, and the supernatant was rotated for 1 h at room temperature. Agarose beads bind to meldonium through the biotin modification and thereby retains proteins complexed with meldonium. After centrifugation at 1,000 × *g* for 3 min and the supernatant was discarded, RIPA buffer (1 ml) was added to the tubes and then rotated for 5 min. Repeated three times. The remaining beads were mixed with the same volume of loading buffer and boiled for 5 min. The eluted proteins were detected by western blotting.

### PFK Activity Assay

PFK activity was measured using a PFK activity colorimetric assay kit (Abcam, Cambridge, United Kingdom) according to the manufacturer’s protocol. Absorbance was measured at 450 nm every 3 min using a microplate reader (Molecular Devices, California, United States). PFK activity is expressed as nmol/min/ml. Experiments were performed at least three times.

### Quantitative Real-Time Polymerase Chain Reaction

Total RNA was extracted from RLE-6TN cells using TRIzol reagent (Invitrogen, Carlsbad, CA), and purified using chloroform, isopropanol, and 75% ethanol. Reverse transcription of cDNA was carried out using a PrimeScript™ RT Reagent Kit with gDNA Eraser (Takara, Osaka, Japan). Quantitative real-time PCR analysis was conducted using a TB Green^®^ Premix Ex Taq™ II (Takara, Osaka, Japan) on a Bio-Rad CFX96 Real-Time PCR Detection System (Bio-Rad Laboratories Ltd., California, United States). Amplification of the products was conducted as follows: 95°C for 30 s, 40 cycles of 95°C for 15 s, 60°C for 30 s, and 72°C for 30 s. A melting curve was delineated using 0.5°C temperature increments every second from 65 to 95°C. The PCR primers were designed using Primer 5.0 software (Premier, Canada), and synthesised by Beijing TsingKe Biotechnology Co., Ltd. (Beijing, China). The primer sequences used were as follows (5ʹ-3ʹ): LDHA Forward: GCCCGCCTGAAGAAGAG, Reverse: GCAGCCTGGACAGTGAA; PFKP Forward: CTCCGCTGTTCGGGTTG, Reverse: GGGTAGGGTGCGTTTCG; PKM2 Forward: ACC​TGG​TGA​CAG​AAG​TGG​A, Reverse: CGGATGAAAGACGCAAA; PDK1 Forward: AAC​TAA​ATG​CGA​AAT​CAC​C, Reverse: TAAACGCCTTTGTCTGC; β-actin Forward: CGC​GAG​TAC​AAC​CTT​CTT​GC, Reverse: CCT​TCT​GAC​CCA​TAC​CCA​CC. Three replicates were used for each sample. Normalisation was performed for β-actin. Relative gene expression was determined using the 2^−ΔΔCt^ method.

### Western Blot Analysis

Cytoplasmic and nuclear proteins in the lung or RLE-6TN cells were extracted using the Cytoplasmic and Nuclear Protein Extraction Reagents Kit (KeyGEN, Nanjing, China) and quantified with a BCA protein reagent kit (KeyGEN, Nanjing, China). Equal amounts of denatured protein were separated by 8% or 10% SDS-PAGE and transferred to PVDF membranes (Millipore, Massachusetts, United States). The membrane was blocked with 5% skim milk at room temperature for 2 h and then incubated with the corresponding primary antibody diluted in blocking buffer at 4°C overnight. Antibodies against PFKP (1:2,000, ab204131), OPA1 (1:2,000, ab42364), and DRP1 (1:2,000, ab184247) were obtained from Abcam (Cambridge, United Kingdom). Antibodies against PKM2 (1:2,000, #4053), LDHA (1:2,000, #2012), PDH (1:2,000, #3205), and β-actin (1:2,000, #4970) were obtained from Cell Signalling Technology (Danvers, MA, United States). The antibodies to Nrf2 (1:2,000, A0674), MFN1 (1:2,000, A9880), MFN2 (1:2,000, A19678), FIS1 (1:2,000, A19666), AQP1 (1:1,000, A15030), AQP4 (1:1,000, A2887), PDK1 (1:2,000, A8930), and lamin B1 (1:2,000, A16909) were obtained from ABclonal (Wuhan, China). Horseradish peroxidase (HRP)-conjugated goat anti-rabbit IgG (H + L) (1:1,000, zb2301) was purchased from ZSGB-BIO (Beijing, China). After incubation with HRP-conjugated secondary antibodies at room temperature for 1 h, a Pro-light HRP Chemiluminescent Kit (TianGen Biotech, Beijing, China) was used to detect the target protein. β-actin and lamin B1 were used as internal controls to normalise the relative expression of each protein. The optical densities of the bands were quantified using the Image Lab analysis software (Bio-Rad, California, United States).

### Statistical Analysis

Data are expressed as the mean ± standard error of the mean. Experimental data were analysed using SPSS Statistics software (version 19.0; IBM Corporation, Armonk, NY, United States) and GraphPad Prism software (Version 8.0, San Diego, CA). Differences among multiple groups were analysed *via* one-way analysis of variance followed by Fisher’s least significant difference test. Statistical significance was set at *p <* 0.05.

## Results

### Meldonium Attenuated Hypoxia-Induced Lung Injury *In Vivo* and *In Vitro*

**FIGURE 1:**
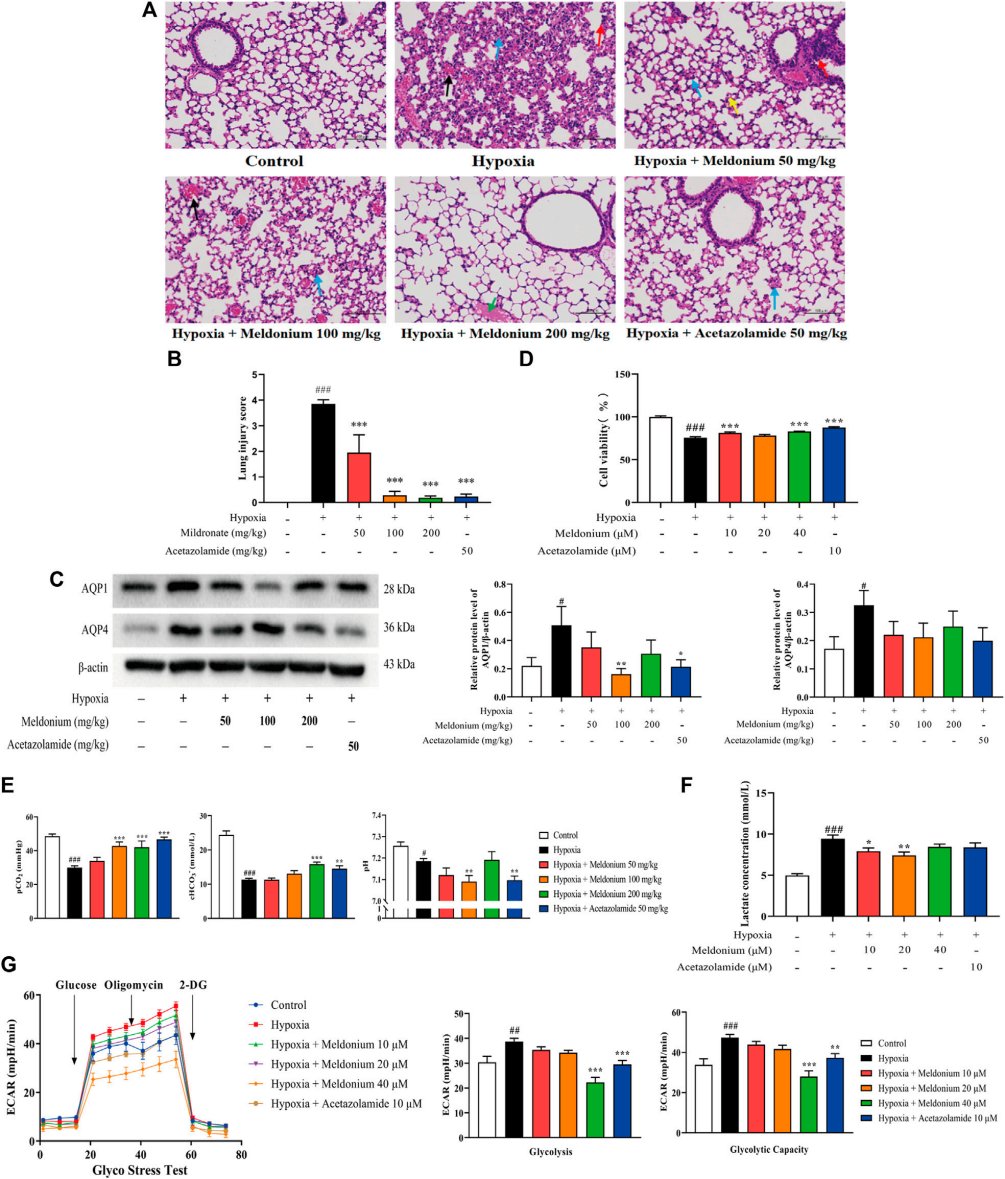
Meldonium treatment attenuated hypoxia-induced lung injury and regulated hypoxia-enhanced glycolysis. **(A)** Histopathology of mouse lungs stained with haematoxylin and eosin (200×, *n* = 7). Black arrowheads indicate haemorrhage, blue arrowheads indicate alveolar wall thickening and alveolar epithelial cell hyperplasia, red arrowheads indicate inflammatory cell infiltration, yellow arrowheads indicate alveolar atrophy, and green arrowheads indicate protein mucus. **(B)** Lung injury score *in vivo* (*n* = 7). **(C)** AQP1 and AQP4 protein expression in lung tissues of lung injury mice (*n* = 7–8). **(D)** Cell viability *in vitro* (*n* = 6). **(E)** Blood gas parameters in mice after hypoxia for 24 h (*n* = 5–11). **(F)** Lactate concentration in the supernatant of cell culture medium *in vitro* (n = 5). **(G)** Extra cellular acidification rate (ECAR) diagram; glycolysis and glycolytic capacity were detected using the Glyco Stress Test Kit *in vitro* (*n* = 5–6). Data are expressed as the mean ± standard error of mean (SEM). Statistical analyses were performed using one-way analysis of variance (ANOVA) followed by Fisher’s least significant difference (LSD) test. #*p* < 0.05, ##*p* < 0.01, ###*p* < 0.001 compared with the control group; *p < 0.05, **p < 0.01, ***p < 0.001 compared with the hypoxia group.

Lung tissue sections stained with H&E (Figure 1A) showed that, compared with the control group, severe lung injury had occurred in mice exposed to hypoxic condition, including thickening of the alveolar walls, infiltration of inflammatory cells, and substantial haemorrhage, indicating that the model of acute exposure to hypoxic conditions was successfully established. Meldonium (50 and 100 mg/kg) modestly attenuated these histological changes. In particular, the meldonium (200 mg/kg) group showed a thin wall structure in the alveolar wall and a nearly complete structure of the alveolar cavity. Furthermore, the lung injury score *in vivo* in the hypoxia group was significantly higher than that in the control group (Figure 1B, *p* < 0.001). However, meldonium (50, 100, and 200 mg/kg) decreased the lung injury score compared with the hypoxia group (*p* < 0.001). Moreover, the expression of aquaporin 1 and 4 (AQP1 and AQP4) was significantly increased compared with the control group *in vivo* (Figure 1C, *p* < 0.05), whereas meldonium (100 mg/kg) markedly decreased AQP1 expression compared with the hypoxia group (100 mg/kg, *p* < 0.01). CCK-8 assay results *in vitro* showed that cell viability was markedly decreased in the hypoxia group compared with the control group (Figure 1D, *p* < 0.001), whereas meldonium (10 and 40 μM) treatment increased cell viability (*p* < 0.01 or *p* < 0.001). Blood gas analysis (Figure 1E) showed that pH, partial pressure of carbon dioxide (pCO_2_), and bicarbonate ion concentration (cHCO_3_^-^) were significantly decreased in the hypoxia group when compared with the control group (*p* < 0.001 or *p* < 0.05), indicating that hypoxia-induced lung injury in mice was accompanied by metabolic acidosis *in vivo*. However, meldonium significantly ameliorated metabolic acidosis by decreasing the level of pCO_2_ (100 and 200 mg/kg, *p* < 0.001) and increasing the level of cHCO_3_^-^ (200 mg/kg, *p* < 0.001) compared to the hypoxia group. Taken together, these findings demonstrate that hypoxia-induced lung injury *in vivo* and *in vitro* can be significantly ameliorated by meldonium pre-treatment, with pharmacodynamic effects similar to acetazolamide.

### Meldonium Regulated Hypoxia-Enhanced Glycolysis *In Vitro*

The lactate concentration data showed that hypoxia increased lactate concentration compared with the control group *in vitro* (Figure 1F, *p* < 0.001), however, meldonium (10, 20, and 40 μM) significantly decreased glycolysis, as indicated by the lower lactate concentration in the cell supernatant compared with the hypoxia group (*p* < 0.001 or *p* < 0.01). Accordingly, the extracellular acidification rate (ECAR) *in vitro* was increased in the hypoxia group compared with the control group, while meldonium reduced the ECAR (Figure 1G). The results of ECAR were analysed in terms of glycolysis, representing the glycolytic ability of cells under basal conditions which was significantly increased in the hypoxia group compared with the control group (*p* < 0.01) and decreased by meldonium (40 μM, *p* < 0.001). Moreover, glycolytic capacity, representing the ability of cells utilising glycolysis to achieve maximum productivity, was significantly increased in the hypoxia group (*p* < 0.001), and decreased by meldonium compared with the hypoxia group (40 μM, *p* < 0.001). Taken together, these findings suggest that meldonium has the ability to regulate glycolysis *in vitro*.

### Target Proteins of Meldonium are Related to Metabolic Pathways

**FIGURE 2:**
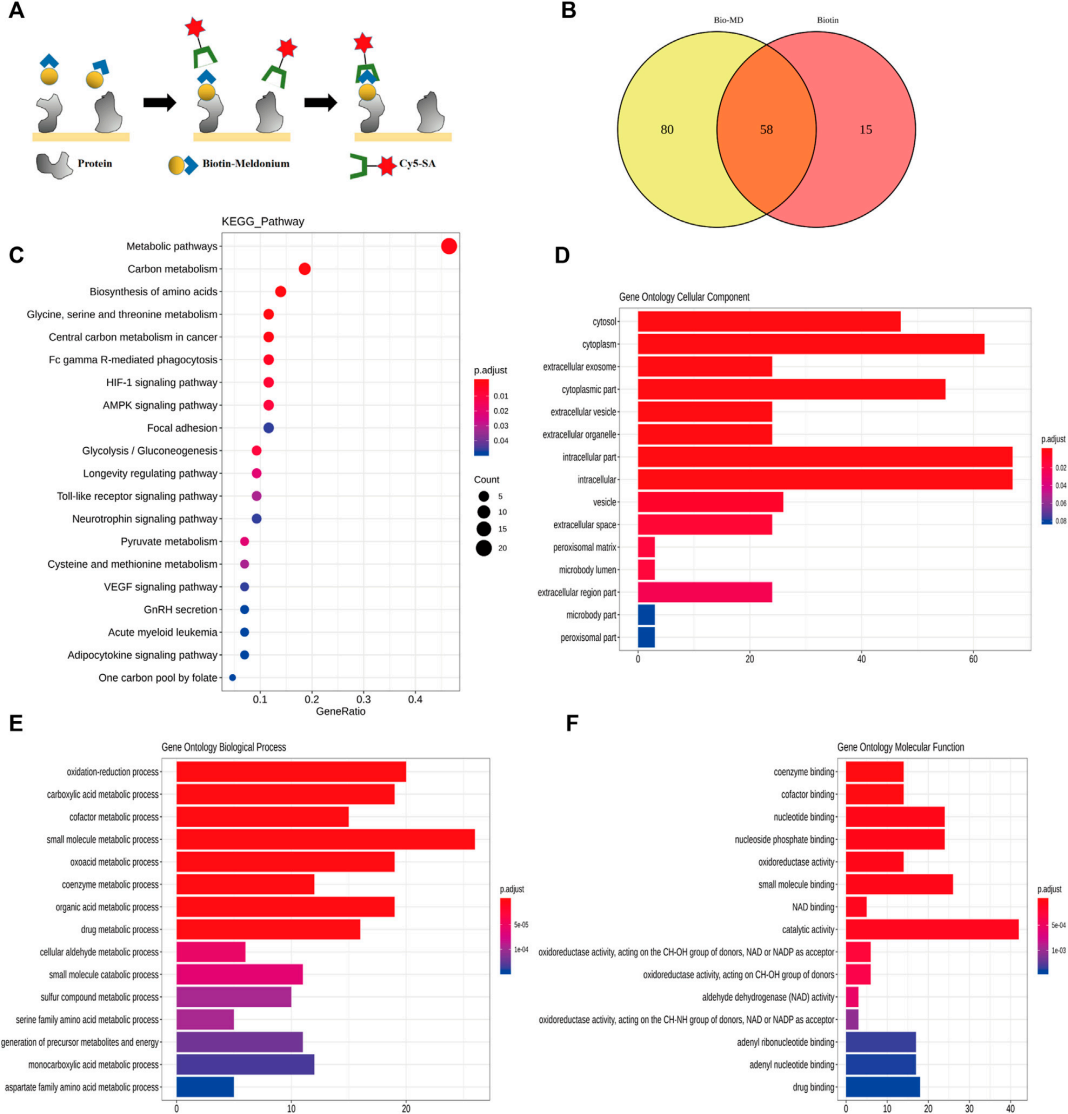
The target proteins of meldonium are related to the metabolic pathway as revealed using the human proteome microarray analysis. **(A)** Schematic of the procedure used to detect biotin-meldonium binding proteins. **(B)** Venn map of the positive spots for meldonium and biotin target proteins. The number in the yellow area represents the number of meldonium-specific target proteins. MD: meldonium. **(C)** Top 20 enriched pathways of meldonium target proteins. **(D)** Top 20 enriched cellular components of meldonium target proteins. **(E)** Top 20 enriched biological processes of meldonium target proteins. **(F)** Top 20 enriched molecular functions of meldonium target proteins.

To confirm the target protein of meldonium, HuProt™ human proteome microarrays assay was performed (Figure 2A). Eighty candidate meldonium target proteins were identified (Figure 2B). The results of the KEGG pathway (Figure 2C) showed that the enriched pathways were mainly involved in energy metabolism, including carbon, glycine, serine, pyruvate and threonine metabolism, glycolysis/gluconeogenesis, and central carbon metabolism. The cellular component indicated that most meldonium target proteins localised to the cytoplasm (Figure 2D). The target proteins were involved in metabolic processes (Figure 2E). Finally, the main function of the target protein was catalysis (Figure 2F). Altogether, the target proteins of meldonium are related to intracellular proteins that have catalytic activity in the metabolic pathways in human cells and require further study to identify.

### Meldonium Regulates Glycolytic Enzymes and Pyruvate Metabolism *In Vivo* and *In Vitro*

**FIGURE 3:**
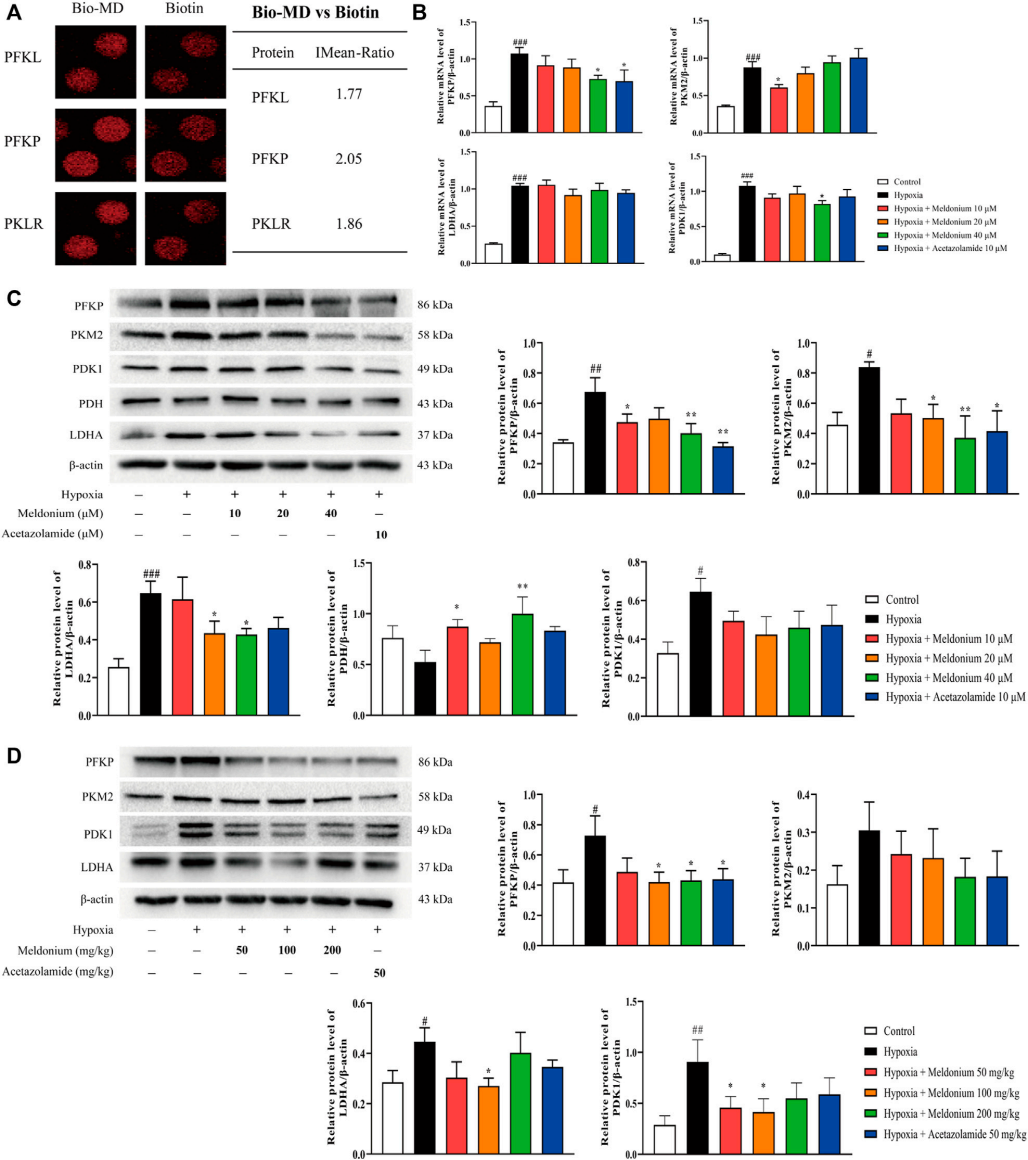
Meldonium regulates glycolytic enzymes and pyruvate metabolism *in vivo* and *in vitro*. **(A)** Representative spots of meldonium target proteins, including the glycolytic process. MD: meldonium. **(B)** mRNA expression of PFKP, PKM2, LDHA, and PDK1 in RLE-6TN cells (*n* = 4–5). **(C)** Protein expression of PFKP, PKM2, LDHA, PDH, and PDK1 in RLE-6TN cells (*n* = 4). **(D)** Protein expression of PFKP, PKM2, LDHA, and PDK1 in lungs of mice after hypoxia for 24 h (*n* = 6–7). Data are expressed as the mean ± SEM. Statistical analyses were performed using one-way ANOVA followed by Fisher’s LSD test. #*p* < 0.05, ##*p* < 0.01, ###*p* < 0.001 compared with the control group; *p < 0.05, **p < 0.01 compared with the hypoxia group.

Consistent with meldonium regulating glycolysis, we found from the human proteome microarray assay that major potential target proteins of meldonium are related to glycolysis (Figure 3A), including PFKP (phosphofructokinase, platelet-type), PFKL (phosphofructokinase, liver type), PKLR (pyruvate kinase, liver, and RBC). The main glycolytic enzymes, including PFKP and M2 isoform of pyruvate kinase (PKM2), catalyse the conversion of glucose to pyruvate and then to lactate *via* the A isozyme of lactate dehydrogenase (LDHA). Pyruvate can also be converted to acetyl coenzyme A by pyruvate dehydrogenase (PDH) in mitochondria, which was regulated by pyruvate dehydrogenase kinase 1 (PDK1), and then participates in aerobic oxidation in mitochondria. These enzymes are involved in glycolysis and pyruvate metabolism. Thus, we first investigated the mRNA expression of enzymes involved in glycolysis and pyruvate metabolism *in vitro*. The mRNA levels of PFKP, PKM2, LDHA, and PDK1 were higher in the hypoxia group than that of the control group (Figure 3B, *p* < 0.001), while meldonium could significantly decrease the mRNA expression of PFKP, PDK1, and PKM2 compared with the hypoxia group (10 or 40 μM, *p* < 0.05).

Accordingly, western blot assay (Figure 3C) showed that the protein expression of PFKP, PKM2, LDHA, and PDK1 was markedly increased after hypoxia exposure *in vitro* (*p* < 0.01 or *p* < 0.05 or *p* < 0.001). But meldonium significantly reduced the protein expression of PFKP, PKM2, and LDHA (10, 20 or 40 μM, *p* < 0.05 or *p* < 0.01) compared with the hypoxia group, while PDK1 was decreased without a significant difference (*p* > 0.05). There were decreases without significant difference (*p* > 0.05) compared with the control group in the protein expression of PDH after exposure to hypoxia, which was significantly increased (*p* < 0.05, *p* < 0.01) by meldonium (10 and 40 μM) compared with the hypoxia group. Additionally, the protein expression of PFKP, LDHA, and PDK1 was significantly enhanced compared with the control group *in vivo* (Figure 3D, *p* < 0.05 or *p* < 0.01), while PKM2 did not show a significant difference (*p* > 0.05). However, meldonium significantly decreased the expression of PFKP, LDHA and PDK1 (10, 20 or 40 μM, *p* < 0.05), while PKM2 did not show a significant difference (*p* > 0.05) compared with the hypoxia group. Western blot results suggested that hypoxia-induced reduction of PDH and increase in PDK1 protein expression were restored by meldonium pre-treatment, which reflected the recovery of aerobic oxidation in mitochondrial energy metabolism by meldonium pre-treatment. Taken together, these results indicate that meldonium has the ability to regulate glycolytic enzymes and mitochondrial metabolic enzymes, which promotes pyruvate metabolism to aerobic oxidation in mitochondria.

### Platelet-Type Phosphofructokinase is the Potential Target Protein of Meldonium

**FIGURE 4:**
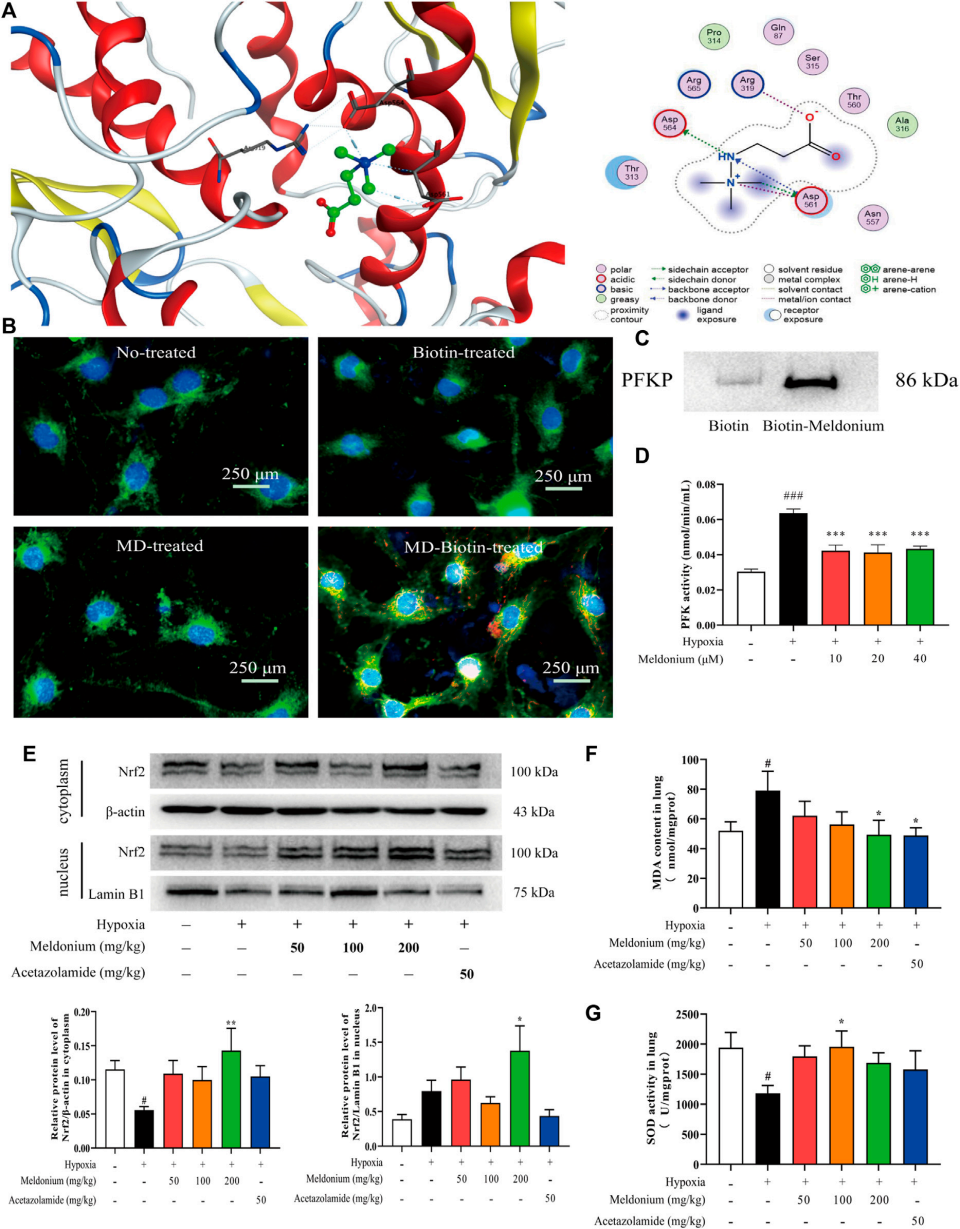
PFKP is the potential target protein of meldonium and meldonium treatment attenuated oxidative stress induced by hypoxia. **(A)** Position of meldonium binding to PFKP and 2D structure diagram of meldonium interacting with PFKP. **(B)** Co-localisation of meldonium with PFKP *in vitro*. PFKP is shown in green, biotin-meldonium is shown in red, and co-localisation is shown in yellow orange. MD: meldonium. **(C)** Pull-down assay of meldonium-PFKP interaction *in vitro* was confirmed *via* western blot. **(D)** Effect of meldonium on PFK activity under hypoxia condition *in vitro* (*n* = 3). **(E)** Protein expression of Nrf2 in the cytoplasm and nucleus after hypoxia for 24 h *in vivo* (*n* = 6). **(F)** Malondialdehyde content after hypoxia for 24 h *in vivo* (*n* = 6). **(G)** Superoxide dismutase activity after hypoxia for 24 h *in vivo* (*n* = 6–7). Data are expressed as the mean ± SEM. Statistical analyses were performed using one-way ANOVA followed by Fisher’s LSD test. #*p* < 0.05, ###*p* < 0.001 compared with the control group; *p < 0.05, **p < 0.01, ***p < 0.001 compared with the hypoxia group.

PFKP is the rate-limiting enzyme of glycolysis and plays a pivotal role in modulating energy metabolism. Consequently, we examined the interaction of meldonium with PFKP protein. First, molecular docking of PFKP with meldonium was performed using MOE software (v2021.09). The binding mode and stability of meldonium with PFKP were analysed by comparing the hydrogen bonds, ionic bonds, and binding energies. The molecular docking results (Figure 4A) show that meldonium forms a hydrogen bond in Asp564 of PFKP and two hydrogen bonds in Asp561 of PFKP. The binding energy was −4.32 kJ/mol, suggesting that meldonium can interact with PFKP protein. After pre-treatment with biotin-labelled meldonium, immunofluorescence experiments showed meldonium co-localisation with PFKP *in vitro* (Figure 4B) in the cytoplasm only. The pull-down assay *in vitro* (Figure 4C) indicated that PFKP protein combined with biotin-labelled meldonium more than the biotin group, suggesting that meldonium has a stable interaction with the PFKP protein. Furthermore, the increase of PFK activity after hypoxia exposure *in vitro* (Figure 4D, *p* < 0.001) was restored by meldonium (10, 20, and 40 μM; *p* < 0.001) compared with the hypoxia group. Collectively, meldonium not only has the potential to bind to PFKP but also regulates PFKP activity. Thus, these results suggest that meldonium regulates glycolysis by targeting PFKP.

### Meldonium Promotes the Translocation of Nrf2 to Attenuate Oxidative Stress Induced by Hypoxia *In Vivo*

We first investigated the expression of Nrf2 in the cytoplasm and nucleus *in vivo* (Figure 4E). There was a notable decrease in Nrf2 protein expression in the cytoplasm after hypoxia compared with the control group *in vivo* (*p* < 0.05), while meldonium (200 mg/kg) significantly increased the expression of Nrf2 compared with the hypoxia group (*p* < 0.01). Furthermore, although not significantly (*p* > 0.05), the protein expression of Nrf2 in the nucleus was increased after hypoxia, which is beneficial to protect against hypoxia-induced oxidative stress. Compared with the hypoxia group, pre-treatment with meldonium (200 mg/kg) significantly increased the protein expression of Nrf2 in the nucleus (*p* > 0.05). These data indicate that meldonium promotes Nrf2 transfer from the cytoplasm to the nucleus *in vivo.*

The changes in oxidative stress-related substances are shown in Figures 4F,G. The level of MDA reflects the degree of lipid peroxidation and SOD, representing the antioxidant capacity. Both indirectly reflect the damage induced by oxidative stress after hypoxia. There was a notable increase in the concentration of MDA (*p* < 0.05), whereas SOD viability was significantly decreased (*p* < 0.05) *in vivo*. However, meldonium significantly decreased MDA content (200 mg/kg, *p* < 0.05) and increased SOD viability (100 mg/kg, *p* < 0.05). Therefore, hypoxia-induced oxidative stress *in vivo* could be ameliorated by meldonium pre-treatment. Overall, these results imply that meldonium promotes the translocation of Nrf2 to the nucleus and resists oxidative stress induced by hypoxia *in vivo*.

### Meldonium Ameliorates Oxidative Stress-Induced Mitochondrial Damage *In Vitro*

**FIGURE 5:**
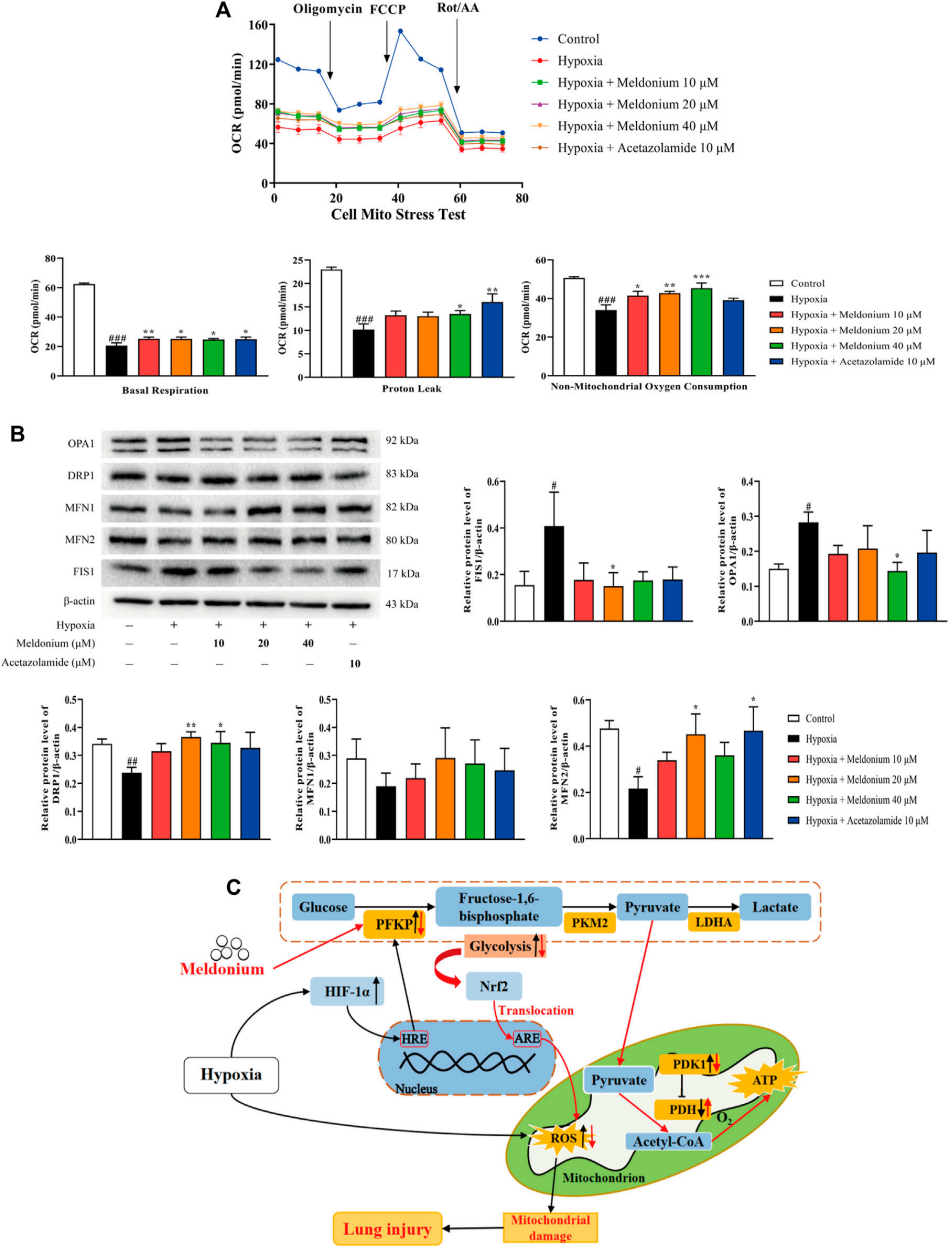
A mechanistic illustration of meldonium amelioration of oxidative stress–induced mitochondrial damage. **(A)** Oxygen consumption rate (OCR) diagram, basal respiration, proton leak, and non-mitochondrial oxygen consumption *in vitro* after hypoxia for 24 h were detected using the Cell Mito Stress Test Kit (n > 3). **(B)** Protein expression of mitochondrial fission and fusion *in vitro* after hypoxia for 24 h were detected *via* western blot (*n* = 3–5). **(C)** A schematic diagram depicting a potential mechanism by which meldonium regulates glycolysis to alleviate hypoxia-induced lung injury. Mechanistically, meldonium can regulate and interact with PFKP to regulate glycolysis that is one of main energy metabolic pathway, while promoting Nrf2 transfer from the cytoplasm to the nucleus. Substantially, Nrf2 actives downstream pathways to prevent oxidative stress and alleviate mitochondrion damage and homeostasis imbalance, which protect the lung from hypoxia-induced injury. Black arrows denote hypoxia-induced changes. Red arrows denote meldonium-induced changes. ARE, antioxidant response elements; HRE, hypoxia response elements; HIF-1α, hypoxia inducible factor-1α; PFKP, platelet isoform of phosphofructokinase; PKM2, M2 type of pyruvate kinase; LDHA, lactate dehydrogenase A; PDH, pyruvate dehydrogenase; PDK1, pyruvate dehydrogenase kinase 1; Nrf2, nuclear factor E2-related factor 2; ROS, reactive oxygen species; ATP, adenosine triphosphate. Data are expressed as the mean ± SEM. Statistical analyses were performed using one-way ANOVA followed by Fisher’s LSD test. #*p* < 0.05, ##*p* < 0.01, ###*p* < 0.001 compared with the control group; *p < 0.05, **p < 0.01, ***p < 0.001 compared with the hypoxia group.

We performed the following experiments to determine whether meldonium has a protective effect on mitochondria after hypoxia *in vitro*. The cell mitochondria stress test *in vitro* (Figure 5A) suggested that cell mitochondrial function was severely affected by hypoxia. The OCR evidently decreased after hypoxia exposure, while meldonium increased OCR compared with the hypoxia group but was lower than the control group. Additionally, various mitochondrial functions were improved. Basal respiration, reflecting the demand for energy production in mitochondria, was obviously decreased after hypoxia compared with the control group (*p* < 0.001), and significantly increased by meldonium compared with the hypoxia group (10, 20, and 40 μM; *p* < 0.01 or *p* < 0.05); Additionally, proton leak, an important parameter of mitochondrial damage, was markedly decreased after hypoxia compared with the control group (*p* < 0.001), while meldonium clearly restored it compared with the hypoxia group (40 μM; *p* < 0.05); Non-mitochondrial oxygen consumption, which reflects the entire condition of cells, was also greatly decreased in the hypoxia group compared with the control group (*p* < 0.001), and obviously increased by meldonium compared with the hypoxia group (10, 20, and 40 μM; *p* < 0.05, *p* < 0.01, and *p* < 0.001).

The homeostasis of mitochondrial fission and fusion that represents the normal state and function of mitochondria is regulated by five proteins. As shown in Figure 5B, after hypoxia *in vitro*, the protein expression of fission protein 1 (FIS1) and optic atrophy 1 (OPA1) significant increased (*p* < 0.05), mitofusins 2 (MFN2) and dynamin-related protein 1 (DRP1) expressions were markedly decreased (*p* < 0.05, *p* < 0.01), while mitofusins 1 (MFN1) levels decreased without any difference (*p* > 0.05) compared with the control group. In contrast, compared with the hypoxia group, meldonium significantly decreased the protein expression of FIS1 and OPA1 (20 or 40 μM, *p* < 0.05), and increased MFN2 (20 μM, *p* < 0.05) and DRP1 expression (20 and 40 μM, *p* < 0.01, *p* < 0.05), whereas MFN1 level increased without significant difference (*p* > 0.05). Thus, we conclude that the damage and dysfunction of mitochondria and imbalance of mitochondrial fission and fusion homeostasis induced by oxidative stress *in vitro* are ameliorated by pre-treatment with meldonium.

## Discussion

This study demonstrated that meldonium markedly ameliorates lung injury induced by hypoxia, providing a new preventive treatment strategy. Mechanistically, by targeting PFKP to regulate glycolysis, which promoting Nrf2 translocation from the cytoplasm to the nucleus, meldonium attenuates hypoxia-induced oxidative stress and adjusts mitochondrial damage. This study is the first to validate the effect of meldonium on hypoxia-induced lung injury under hypobaric hypoxic conditions. It clarifies the possible molecular mechanism of action involved, providing an opportunity for a drug support strategy of low altitude residents acute exposed to hypobaric hypoxic conditions.

Hypoxia at high altitudes is the main cause of AMS, such as HACE and HAPE. Travellers or workers visit high-altitude regions and participate in activities that may cause hyposthenia without specialised training, resulting in fatigue and decreased immunity, and even respiratory diseases. Previous studies have demonstrated that treatment with meldonium for long time (100, 200 and 400 mg/kg) does not cause pathological damage to liver or heart (Hayashi et al., 2000; Liepinsh et al., 2009). In this study, we first investigated the preventive effects of meldonium on hypoxia-induced lung injury. In our animal experiments simulating acute exposure to hypobaric hypoxic conditions, hypoxia-induced severe lung injury *in vivo*, such as the significant pathological changes of lung tissue, could be alleviated by pre-treatment with meldonium. A recent study has demonstrated that meldonium treatment mitigates the development of cardiovascular complications and has potential applicability in treating patients with COVID-19 (Vilskersts et al., 2021). According, lung injury caused by hypobaric hypoxic conditions is similar to COVID-19, and meldonium maybe has the potential to alleviate relevant symptoms. Previous studies have demonstrated that the expression of AQP1 is related to lung injury induced by pancreatitis and oleic acid (Zhu et al., 2008; Liu et al., 2014). In high-altitude environments, hypoxia leads to changes in AQP1, resulting in abnormal transmembrane transport of water molecules in the lungs (Zhou et al., 1993). Thus, AQP can be used as a biomarker for lung injury. Here, the protein expression of AQP1 was significantly increased *in vivo* after exposure to hypoxia and could be alleviated by pre-treatment with meldonium. Additionally, blood gas indices showed that metabolic acidosis induced by hypoxia, a marker of lung injury (Zhou et al., 1993), was alleviated by pre-treatment with meldonium. Furthermore, under hypoxic condition, alveolar epithelial cells (AECs) are extremely prone to induce apoptosis which is a marker of lung injury, and netrin-1 alleviates hypoxia-induced lung injury by reducing alveolar epithelial cell apoptosis (Ko et al., 2019). In this study, we found that meldonium improved type II AEC viability after hypoxia *in vitro*. Taken together, these data indicate that pre-treatment with meldonium has the potential to ameliorate hypoxia-induced lung injury under hypobaric hypoxic conditions. However, the mechanism needs further investigation.

A special environment, such as hypobaric hypoxic high-altitude conditions, also is a key factor influencing the functional metabolic phenotype (Chang et al., 2019). Cells respond to hypoxia through the HIF-1 signalling pathway (Bunn and Poyton, 1996). Glycolysis is an essential metabolic pathway regulated by various enzymes, which are regulated by HIF-1α under hypoxic condition. A recent study reported that the excessive enhancement of glycolysis induced lung injury (Prasad et al., 2017). Besides, the increased lactate concentration in blood from glycolysis is a clear symptom in hypoxic environments, and excessive lactate induces lung injury (Pourfathi et al., 2020). Researches showed that meldonium reduces L-carnitine content, modulates myocardial energy metabolism and hepatic lipid metabolism in rats in a dose-dependent manner (Hayashi et al., 2000; Liepinsh et al., 2009), but without glycolysis. In this study, hypoxia induced the significant increase in glycolysis levels and lactate concentration *in vitro*. Together with the occurrence of lung injury in mice after exposure to hypobaric hypoxic conditions, there may be a mechanistic relationship between glycolysis and lung injury. Further research revealed that ALI induced by lipopolysaccharide (LPS) was alleviated by a glycolysis inhibitor (Zhong et al., 2019), and was ameliorated by inhibiting PFKFB3, an allosteric activator of PFK-1 (Wang et al., 2019). Thus, regulating glycolysis is an effective strategy to alleviate lung injury. Given the action of meldonium ameliorating hypoxia-induced lung injury, we further investigated whether meldonium could regulate glycolysis. In our study, meldonium pre-treatment significantly decreased the enhancement of real-time glycolysis and lactate concentration *in vitro* following hypoxia. These results suggest that meldonium has the ability to regulate glycolysis, the key to ameliorating hypoxia-induced lung injury.

However, glycolysis is mediated by several enzymes and can be divided into two stages, the glycolytic pathway, which catalyses the conversion of glucose to pyruvate, and the reduction of pyruvate to lactate without oxygen consumption. To further elucidate the mechanism of action of meldonium and confirm its target protein, Human Proteome Microarray Assay was performed. The results indicated that the target protein of meldonium is closely associated with intracellular proteins with catalytic activities in metabolic pathways. Based on further results, the main target protein candidates of meldonium are the enzymes of glycolysis, including PFKP, PFKL, and PKLR. These data further substantiate the conclusion that meldonium alleviates hypoxia-induced lung injury by regulating glycolysis. Indeed, previous research has demonstrated that meldonium regulates glucose metabolism (Liepinsh et al., 2008), however, these studies neglected glycolysis and related enzymes. In this study, we further screened potential target proteins and found that the mRNA and protein expression of important glycolytic enzymes and pyruvate metabolic enzymes, such as PFKP, PKM2, LDHA, and PDK1, were significantly increased, and that of PDH was decreased after hypoxia, while meldonium modulated these changes. These results suggested that meldonium can regulate glycolytic enzymes and promote pyruvate metabolism to aerobic oxidation. Together with pre-treatment with meldonium decrease lactate concentration after hypoxia, this study further clarifies the mechanism by which meldonium regulates glucose metabolism—by promoting the aerobic oxidation of pyruvate after the glycolytic pathway without glycolysis that with lactate as the end product. Taken together, these data indicated that meldonium regulates glycolysis to alleviate hypoxia-induced lung injury by targeting glycolytic enzymes. More importantly, the mRNA and protein expression of PFKP was particularly affected by meldonium, and PFKP is likely to be the target protein of meldonium; however, further research is warranted to confirm this hypothesis.

Glycolysis is highly susceptible to hypoxia, and is regulated by various glycolytic enzymes (Mittal et al., 2014). A previous study suggested that the activity of PFK, a rate-limiting enzyme of glycolysis, was increased in chronic obstructive pulmonary disease and under hypoxic condition, which consequently influences the respiratory system (Simon et al., 1981). PFKP, the platelet type of PFK, influences the respiratory system by regulating glycolysis (Park et al., 2020) and plays a central role in the progression of pulmonary disease under hypoxic condition (Zhang et al., 2021). These reports indicate that the glycolytic enzyme PFKP may be involved in the mechanism of action of meldonium for alleviating hypoxia-induced lung injury under hypobaric hypoxic conditions. Thus, together with the hypothesis that PFKP is the potential target protein of meldonium, we focused on the PFKP protein for further investigation. In this study, the molecular docking results indicated that meldonium can interact with the PFKP protein. Besides, the co-localisation of meldonium and PFKP was found in cytoplasm using immunofluorescence, which suggested that meldonium may be combined with PFKP protein. The result of the pull-down assay revealed that the protein expression of PFKP in the biotin-meldonium group was higher than that in the biotin group, which prompted that the stable interaction between meldonium and PFKP protein. Additionally, meldonium regulates PFK activity under hypoxic condition. Thus, these results indicated that meldonium has the potential to stably bind to and interact with the PFKP protein, which further corroborates that meldonium regulates glycolysis by targeting the PFKP protein to alleviate hypoxia-induced lung injury. Compared with previous study (Liepinsh et al., 2008), we further clarify meldonium regulates glucose metabolism by targeting glycolysis enzyme-PFKP.

Apart from glycolysis, oxidative stress is also an important factor in hypobaric hypoxia-induced lung injury. Mechanistically, the excessive production of ROS in lung tissue induced by hypobaric hypoxia leads to oxidative stress, which plays a role in acute lung injury (Klimova and Chandel, 2008). In this study, MDA levels were increased and SOD levels decreased *in vivo* in hypoxia-induced lung injury mice, both indirectly reflecting the extent of oxidative stress. Pre-treatment with meldonium significantly reversed these changes, suggesting that the anti-oxidative stress effect of meldonium was also involved in alleviating hypoxia-induced lung injury. Moreover, Nrf2, a regulatory factor in the nucleus, activates the transcription of genes that have antioxidant response element (ARE) at the promoter site to protect cells from oxidative stress injury in high-altitude conditions (Gong et al., 2018), which is crucial in reducing oxidative stress. Consistent with this report, we found that meldonium promoted the translocation of Nrf2 into the nucleus under hypoxic condition *in vivo* in our study. Besides, there is a negative correlation between glycolysis and Nrf2 (Cai et al., 2020), and inhibiting glycolysis promotes Nrf2 translocation from the cytoplasm to the nucleus (Bollong et al., 2018). Thus, glycolysis plays a leading role in regulating Nrf2 and oxidative stress. Together, our study showed that meldonium protects from hypoxia-induced lung injury by targeting the glycolytic enzyme PFKP to regulate glycolysis, which promoted the translocation of Nrf2 into the nucleus and alleviated oxidative stress under hypoxic condition.

Further, research has shown that imbalance or damage to mitochondrial dynamic homeostasis is a major contributor to lung injury caused by oxidative stress (Zhang et al., 2019). The homeostatic imbalance of mitochondrial fission and fusion is mediated by relevant proteins including DRP1, FIS1, MFN1, MFN2, and OPA1, and constitute the principal manifestation of an oxidative stress injury in cells caused by environmental stimulation (Klimova and Chandel, 2008). In this study, meldonium pre-treatment significantly reversed the changes in the protein expression of DRP1, FIS1, MFN2, and OPA1 *in vivo* under hypobaric hypoxic conditions, suggesting that meldonium could redress the imbalance of mitochondrial homeostasis induced by oxidative stress. Additionally, the mitochondrial function and status results showed that hypoxia induced a reduction in basal respiration, proton leak and non-mitochondrial oxygen consumption were increased following meldonium pre-treatment, which indicated that meldonium ameliorated mitochondrial damage from oxidative stress. These data suggest that meldonium has the ability to protect mitochondria from oxidative stress, further explaining the protective effect of meldonium in the lung under hypoxic condition from an oxidative stress perspective.

This study has some limitations. Biologically, PFKP crystallises as a tetrameric structure. Residues in the tetrameric interface influence tetramer formation, enzyme catalytic capacity, and regulation. Further research should be conducted to investigate the interaction points in the structure of meldonium drugs and PFKP proteins. In addition, regulating energy metabolism in the mitochondria is one of the important roles of meldonium. In future research, we plan to explore the mechanism by which meldonium ameliorates aerobic oxidation in the tricarboxylic acid cycle to meet the demand for ATP in extreme environments.

In conclusion, the findings of this study indicate that meldonium pre-treatment alleviated hypoxia-induced lung injury under hypobaric hypoxic conditions *in vivo* and *in vitro*. The present study identifies a potential novel pre-treatment drug to prevent AMS. Our results also clarify the underlying mechanism of action of meldonium. Meldonium targets PFKP to regulate glycolysis, which promotes Nrf2 translocation to the nucleus against hypoxia-induced oxidative stress and mitochondrial damage (Figure 5C). We identify the PFKP protein as the key target of anti-hypoxia lung injury at hypobaric hypoxic conditions or high altitudes, which has laid an important research foundation for follow-up clinical drug research and development and precision treatment. Moreover, we confirm that promoting pyruvate metabolism to aerobic oxidation after the glycolytic pathway is the mechanism by which meldonium regulates glucose metabolism. Thus, future studies should focus on the effect of meldonium to regulate energy metabolism in mitochondria.

## Data Availability

The original contributions presented in the study are included in the article/Supplementary Material, further inquiries can be directed to the corresponding authors.
